# A participatory supportive return to work program for workers without an employment contract, sick-listed due to a common mental disorder: an economic evaluation alongside a randomized controlled trial

**DOI:** 10.1186/s12889-017-4079-0

**Published:** 2017-02-02

**Authors:** Lieke Lammerts, Johanna M. van Dongen, Frederieke G. Schaafsma, Willem van Mechelen, Johannes R. Anema

**Affiliations:** 1 Department of Public and Occupational Health, EMGO+ Institute for Health and Care Research, VU University Medical Center, Van der Boechorststraat 7, Amsterdam, NL-1081 BT The Netherlands; 2 Research Center for Insurance Medicine AMC-UMCG-UWV-VUmc, Amsterdam, The Netherlands; 3 Department of Health Sciences, EMGO+ Institute for Health and Care Research, Faculty of Earth & Life Sciences, Vrije Universiteit Amsterdam, Amsterdam, The Netherlands

**Keywords:** Cost-effectiveness evaluation, Cost-utility evaluation, Return on investment, Randomized controlled trial, Societal perspective, Social insurer’s perspective, Return to work, Occupational healthcare, Common mental disorders, Vulnerable workers

## Abstract

**Background:**

Mental disorders are associated with high costs for productivity loss, sickness absence and unemployment. A participatory supportive return to work (RTW) program was developed in order to improve RTW among workers without an employment contract, sick-listed due to a common mental disorder. The program contained a participatory approach, integrated care and direct placement in a competitive job. The aim of this study was to evaluate the cost-effectiveness and cost-utility of this new program, compared to usual care. In addition, its return on investment was evaluated.

**Methods:**

An economic evaluation was conducted alongside a 12-month randomized controlled trial. A total of 186 participants was randomly allocated to the new program (*n* = 94) or to usual care (*n* = 92). Effect measures were the duration until sustainable RTW in competitive employment and quality-adjusted life years (QALYs) gained. Costs included intervention costs, medical costs and absenteeism costs. Registered data of the Dutch Social Security Agency were used to assess the duration until sustainable RTW, intervention costs and absenteeism costs. QALYs and medical costs were assessed using three- or six-monthly questionnaires. Missing data were imputed using multiple imputations. Cost-effectiveness analysis and cost-utility analysis were conducted from the societal perspective. A return on investment analysis was conducted from the social insurer’s perspective. Various sensitivity analyses were performed to assess the robustness of the results.

**Results:**

The new program had no significant effect on the duration until sustainable RTW and QALYs gained. Intervention costs and medical costs were significantly higher in the intervention group. From the societal perspective, the maximum probability of cost-effectiveness for duration until sustainable RTW was 0.64 at a willingness to pay of about €10 000/day, and 0.27 for QALYs gained, regardless of the willingness to pay. From the social insurer’s perspective, the probability of financial return was 0.18.

**Conclusions:**

From the societal perspective, the new program was neither cost-effective in improving sustainable RTW nor in gaining QALYs. From the social insurer’s perspective, the program did not result in a positive financial return. Therefore, the present study provided no evidence to support its implementation.

**Trial registration:**

The trial was listed at the Dutch Trial Register (NTR) under NTR3563 on August 7, 2012.

**Electronic supplementary material:**

The online version of this article (doi:10.1186/s12889-017-4079-0) contains supplementary material, which is available to authorized users.

## Background

Mental disorders are associated with high costs for the individuals concerned, employers, the social security system, and society as a whole. In Europe, the overall cost of mental disorders is estimated at 3–4% of the gross domestic product [1]. The majority of these costs is made outside the healthcare sector and is related to loss of potential labor supply, sickness absence, reduced productivity at work, and unemployment [1, 2]. To illustrate, individuals with a severe mental disorder are 6 to 7 times less likely to be employed compared with individuals without such a disorder. The risk of unemployment is smaller when disorders are milder. Nevertheless, individuals with mild to moderate mental disorders, also known as common mental disorders (CMDs), are still 2 to 3 times less likely to be employed [1]. This is an important concern, as mental disorders are highly prevalent in the working-age population and around three quarters of those affected by a mental disorder have a CMD [1]. Moreover, several Dutch studies comparing sick-listed workers without an employment contract with sick-listed employees revealed that the former often experience a worse health status and face more obstacles for return to work (RTW) [3]. As a result, these workers have an increased risk for long-term disability [4].

Despite the aforementioned association between mental ill health and unemployment, until now most intervention research aiming to improve work participation of workers sick-listed due to a CMD have assumed the presence of a workplace [5]. For this reason, the participatory supportive RTW program was developed. The aim of this program was to shorten the duration until RTW of workers without an employment contract who are sick-listed due to a CMD. This program was based on three best practices in occupational healthcare (OHC): a participatory approach, integrated care, and direct placement in a competitive job [6].

We evaluated the effectiveness of this new participatory supportive RTW program on the duration until sustainable RTW, in comparison with usual OHC in the Netherlands for sick-listed workers without an employment contract [7]. However, to make a business case for or against the intervention it is also important to evaluate the (additional) societal cost per unit of effect gained [8]. As decision-makers are often confronted with limited resources, they need to decide on the optimal allocation of resources to maximize a certain desired outcome or benefit [9]. Therefore, the aim of the present study was to conduct a cost-effectiveness analysis (CEA) and a cost-utility analysis (CUA) to assess the (additional) societal costs per one day earlier sustainable RTW and per quality-adjusted life years (QALYs) gained. In addition, we evaluated the financial return on investment (ROI) from the social insurer’s perspective. In the Netherlands the Dutch Social Security Agency (SSA) is responsible for sickness benefit payment and OHC for sick-listed workers without an employment contract. Because of this, the SSA is interested in the financial return of the new program. Our main research question was: what was the cost-effectiveness and cost-utility of the new participatory supportive RTW program from the societal perspective, in comparison with usual OHC? A second research question was: what was the ROI of the new program, compared to usual OHC, from the social insurer’s perspective?

## Methods

### Study population and design

An economic evaluation was conducted alongside a 12-month randomized controlled trial (RCT), titled ‘The Co-WORK study’, which took place between 2013 and 2015. The trial was carried out in collaboration with seven offices of the Dutch SSA, located in three districts, and with three vocational rehabilitation agencies. Participants were recruited via an invitational letter from the medical advisor of the Dutch SSA. This invitational letter was sent one to two weeks after sick-listing, so that an early intervention could take place. This has been considered important in the prevention of long-term sickness absence [10]. Because it was not possible to recruit on the basis of a registered mental health complaint, every newly sick-listed worker received an invitation. Sick-listed workers were asked to only respond to this invitation when they experienced mental health problems. Eligible for participation were workers sick-listed between two and 14 weeks, who had applied for a sickness benefit at the Dutch SSA due to the (partial) absence of an employment contract, with mental health problems as the main reason for their claim. Other inclusion criteria were: an elevated level of distress, based on a distress screener [11], and the intention to return to work despite ongoing health complaints, as workers without this intention less likely seem to benefit from a participatory approach [12, 13].

Participants were randomly allocated to an intervention or control group, after they had completed the baseline questionnaire. Before randomization, pre-stratification took place based on type of worker before sick-listing (i.e. unemployed worker, temporary agency worker, and fixed-term contract worker) and the three participating SSA districts. Schemes with random permuted numbers were used to create a block randomization table for each stratum, with fixed block sizes of four. A research assistant performed the randomization during an intake meeting with the participant. Due to the nature of the intervention, blinding participants and professionals for the randomization result was not possible.

More information about the study design and setting, in- and exclusion criteria for participation, recruitment procedures, randomization and blinding, and the sample size calculation can be found in the study protocol [6].

### Interventions

#### Usual occupational healthcare

Usually, OHC is provided by a team of professionals from the SSA consisting of an insurance physician, a labor expert and a RTW coordinator. The OHC starts with an examination of the sickness benefit claim by the RTW coordinator. Subsequently, the RTW coordinator, the insurance physician and the labor expert together decide whether it is necessary for the sick-listed worker to visit the insurance physician and/or the labor expert for (medical) examination and/or advice on recovery and RTW. If necessary, the sick-listed worker can be referred to work disability oriented treatment or additional vocational rehabilitation support. The OHC that is actually delivered to the sick-listed worker is dependent on the sick-listed worker’s progress in vocational rehabilitation.

#### The participatory supportive RTW program

All participants were entitled to usual OHC. In addition, participants in the intervention group were referred to a more protocolled form of OHC that started early after sick-listing and contained several best practices. Two of these practices, i.e. application of a participatory approach and integrated care, were new and one of these practices, i.e. placement in a competitive job by a vocational rehabilitation agency, is also possible in usual OHC, but has not been protocolled. Table 1 briefly describes the content of this participatory supportive RTW program. A more detailed description is presented in the study protocol [6]. Participating professionals were trained in the execution of this program during one session of approximately two and a half hours by the researchers, by means of a presentation and role plays. Training sessions were organized for each SSA office or district separately. In addition, follow-up sessions were planned at each SSA office, to evaluate the first cases and to discuss difficulties in applying the program in daily practice.

**Table 1:** The participatory supportive RTW program

Examination of sickness benefit claim and medical problem analysis – *within two weeks after allocation to the intervention team*
- The RTW coordinator examines the sickness benefit claim, conform usual care
- The insurance physician makes a medical problem analysis, conform usual care
Integrated care – *directly after the medical problem analysis*
- The insurance physician contacts the healthcare provider(s) of the sick-listed worker by telephone to agree upon treatment and RTW, and to stimulate cooperation during the vocational rehabilitation process
Participatory approach – *within two weeks after the medical problem analysis*
- The labor expert supports the sick-listed worker and the RTW coordinator separately in identifying and prioritizing obstacles for RTW
- The sick-listed worker and the RTW coordinator jointly search for solutions to overcome the main obstacles for RTW, and discuss suitable work
- The labor expert tries to reach consensus between the sick-listed worker and the RTW coordinator, and summarizes the consensus-based solutions and suggestions for suitable work in a RTW action plan
- The insurance physician makes adjustments to the RTW action plan, if necessary
- The labor expert sends the final action plan to all stakeholders involved, and underlines the sick-listed worker’s own responsibility to implement the action plan
Direct placement in a competitive job – *within four weeks after making a RTW action plan*
- The RTW coordinator refers the sick-listed worker to a vocational rehabilitation agency to facilitate the job search
- The vocational rehabilitation agency offers the sick-listed worker at least two suitable competitive workplaces with a minimum contract period of 3 months, matching the RTW action plan
- The sick-listed worker is placed in a suitable competitive workplace
Evaluation – *within four weeks after making a RTW action plan*
- The RTW coordinator contacts the sick-listed worker to evaluate the implementation of the RTW action plan
- The RTW coordinator contacts the vocational rehabilitation agency to inquire if the sick-listed worker has been placed in a suitable workplace
- The sick-listed worker is referred to two other vocational rehabilitation agencies for additional vocational support, if necessary

### Effect measures

#### Duration until sustainable RTW

The primary effect measure was duration until sustainable RTW in a competitive job, defined as the duration in calendar days from the day of randomization until (partial) work resumption for at least 28 calendar days in a regular work setting for which payment is received at the market rate [6]. When the participant was only partially sick-listed, he was considered to have reached the outcome when he had (partially) returned to work for the hours he/she had been sick-listed for. For participants who had not reached the outcome, the total follow-up time of 365 days was taken into account.

Data about paid employment is registered continuously by the Dutch SSA, and was collected from this database after one year follow-up. Every three months, starting at baseline, questionnaires were used to collect additional data on work resumption, to facilitate interpretation of the registered data.

#### Quality-adjusted life years

At baseline, and after 6 and 12 months, the EuroQol-5D-3 L [14] was used to assess quality of life. Scores on the five ‘health dimension’ items (range 1–3) in this questionnaire were translated into a utility score (on a scale of 0–1, from equal to death to equal to full health) using the Dutch tariff [14]. QALYs were calculated by multiplying the obtained utility scores with the duration in this health state, using linear interpolation between measurement points.

### Resource use and valuation

#### Intervention costs

Table 2 gives an overview of the applied cost categories and corresponding unit prices for determining intervention costs. Data on applied OHC during follow-up (i.e. the number of consults with professionals from the Dutch SSA and referrals by the SSA for additional support) were obtained from the SSA database. Consults were valued using labor costs (including overhead) of the SSA professionals. Costs for additional support were valued using market prices, which were obtained from the SSA database as well.

**Table 2:** Assessment of intervention costs

Cost categories		Unit prices
Applied OHC by the Dutch SSA – Intervention and control group		
Number of consults with OHC professionals from the Dutch SSA	- RTW coordinator	€58.50/h
	- Insurance physician	€106.20/h
	- Labor expert	€80.60/h
Referrals by the SSA to work disability oriented treatment or additional vocational rehabilitation support		Market price
Training in the participatory supportive RTW program – Intervention group		
Number of hours attending the training	- RTW coordinator	€58.50/h
	- Insurance physician	€106.20/h
	- Labor expert	€80.60/h
Number of hours providing the training	- Junior researcher	€33.30/h
	- Senior researcher	€67.90/h
	- Professor	€124.90/h

Costs for the training in the participatory supportive RTW program were estimated using data on the number and duration of provided training sessions, as well as labor costs (including overhead) of SSA professionals attending the sessions, and the researchers providing the sessions.

#### Medical costs

Medical costs were assessed every three months using The Trimbos/iMTA questionnaire for costs associated with psychiatric illness (Tic-P) [15], measuring resource use with a 3-month recall period. The questionnaire included primary healthcare (i.e. consults with a general practitioner, allied healthcare, and complementary medicine), secondary healthcare (i.e. specialized healthcare, and hospitalization) and the use of medication. As for the use of medication, only the use of psychotropics, excluding antipsychotics, was included. Healthcare utilization was valued using Dutch Standard Costs [16] or, if not available, prices according to the professional organizations. Prices provided by the Dutch Society of Pharmacy [17] were used to value medication use.

#### Absenteeism costs

From the societal perspective, absenteeism costs were estimated by considering productivity loss. Because all participants were (partially) unemployed at the time of sick-listing, productivity loss could not be estimated based on sickness absence days from work. Instead, our starting point was the maximum number of productive hours for a Dutch employee in full-time employment (36 h/week), accounting for holidays and other days off, which was 1540 h per year [16]. This number was regarded as the maximally possible productivity loss. The participants’ level of productivity loss was estimated by subtracting the total number of hours in paid employment during follow-up, obtained from the SSA database, from the aforementioned maximum duration of productivity loss. We used the Human Capital Approach (HCA) to value productivity loss, by multiplying the loss of productivity in hours by the estimated price of productivity loss for a Dutch worker per hour, based on sex and age [16].

From the social insurer’s perspective, absenteeism costs were calculated using the real costs for sickness benefit and employment benefit payment during follow-up, obtained from the SSA database.

All costs were converted to 2014 Euros using consumer price indices [18]. As the follow-up of the trial was one year, discounting of costs and effects was not necessary.

### Statistical analyses

Analyses were performed according to the intention-to-treat principle. Statistical significance was set at *p* < 0.05. Data were analyzed using Stata (V12, Stata Corp, College Station, TX). Descriptive statistics were used to compare baseline characteristics between the intervention and control group, as well as between participants with complete and incomplete data. Missing values for costs and effects were imputed separately for the intervention and control group using multiple imputations, through Predictive Mean matching. In total, five complete datasets were created (loss of efficiency ≤ 5%) [19]. All of the imputed datasets were analyzed as specified below, after which pooled estimates were calculated using Rubin’s rules [20].

#### Societal perspective: cost-effectiveness and cost-utility analyses

The CEA and CUA were conducted from the societal perspective, which means that all costs related to the intervention were taken into account irrespective of who pays or benefits. However, absenteeism costs were excluded from the CEA, as these costs could be considered as a proxy for the effect measure (i.e. time to sustainable RTW). Effect differences, in terms of duration until sustainable RTW and QALYs, and cost differences between the intervention and control group were analyzed simultaneously using seemingly unrelated regression (SUR). Hereby, cost and effect difference estimates could be adjusted for their possible correlation [21]. Furthermore, all estimates were corrected for possible prognostic factors for RTW as identified in the existing literature, i.e. demographic characteristics, type of worker before sick-listing, RTW expectation, RTW intention, fear avoidance beliefs, and Attitude, Social influence and self-Efficacy (ASE) regarding RTW [12, 13, 22–24]. All these possible prognostic measures were assessed at baseline. Because of the skewness of the cost data, 95% confidence intervals (CIs) surrounding the cost-differences were estimated using bias-corrected and accelerated (BCA) bootstrap intervals, with 5000 replications. Subsequently, incremental cost-effectiveness ratios (ICERs) were calculated by dividing the corrected mean cost differences by those in effects. By plotting bootstrapped incremental cost-effect pairs (CE-pairs) on cost-effectiveness planes (CE-planes), the uncertainty surrounding the ICERs was graphically illustrated [25]. Cost-effectiveness acceptability curves (CEACs) were plotted, presenting the intervention’s probability of cost-effectiveness at different values of willingness to pay [26].

#### Social insurer’s perspective: ROI analyses

ROI analyses were performed from the social insurer’s perspective. Costs were defined as the mean difference in intervention costs (i.e. differences in costs for applied OHC and costs for the training in the participatory supportive RTW program). Benefits were defined as the mean difference in absenteeism costs between the intervention and control group (i.e. the mean difference in paid sickness benefits and unemployment benefits). Positive benefits indicated reduced spending. Costs and benefits were estimated using SUR analyses, and corrected for the same possible confounders as described above. The 95% CIs surrounding costs and benefits were estimated using bias-corrected and accelerated (BCA) bootstrap intervals, with 5000 replications. Three ROI-metrics were calculated; 1) Net Benefits (NB), 2) Benefit Cost Ratio (BCR), and 3) Return on Investment (ROI).

$$ N B= B e n e f i t s\hbox{--} C o s t s; B C R= B e n e f i t s/ C o s t s; R O I=(( B e n e f i t s\hbox{--} C o s t s)/ C o s t s)\ast 100 $$

Financial returns are positive when NB > 0, BCR > 1, and ROI > 0% [27–29]. To quantify the precision of these metrics, 95% bootstrapped confidence intervals were estimated, using 5000 replications. Subsequently, the probability of financial return was estimated based on the proportion of bootstrapped NBs, BCRs, and/or ROIs, indicating cost savings [30].

### Sensitivity analyses

To assess the robustness of the results, five sensitivity analyses (SA) were performed. First, analyses were performed using the complete-cases only (SA1). Second, analyses were performed excluding healthcare outliers, i.e. cases in which expenses for secondary care were above €10 000 (SA2). Third, per-protocol analyses were performed, comparing intervention group participants who had actually started with the participatory supportive RTW program after the medical assessment with control group participants (SA3). Finally, for the CEA and CUA only, two additional sensitivity analyses were performed in which the Friction Cost Approach (FCA) was used instead of the HCA to value productivity loss. According to this approach, organizations need a certain period to replace a sick-listed worker (i.e. friction period). When a sick-listed worker is replaced, productivity loss stops. In the Netherlands, the estimated friction period was assumed to be 23 weeks [16]. More recently, however, a friction period of 12 weeks has been assumed [31]. We accounted for both friction periods, which means that when productivity loss exceeded the friction period of 23 or 12 weeks, costs were truncated at the costs of 23 or 12 weeks of productivity loss in SA4 and SA5, respectively.

## Results

### Participants

**Fig. 1:**
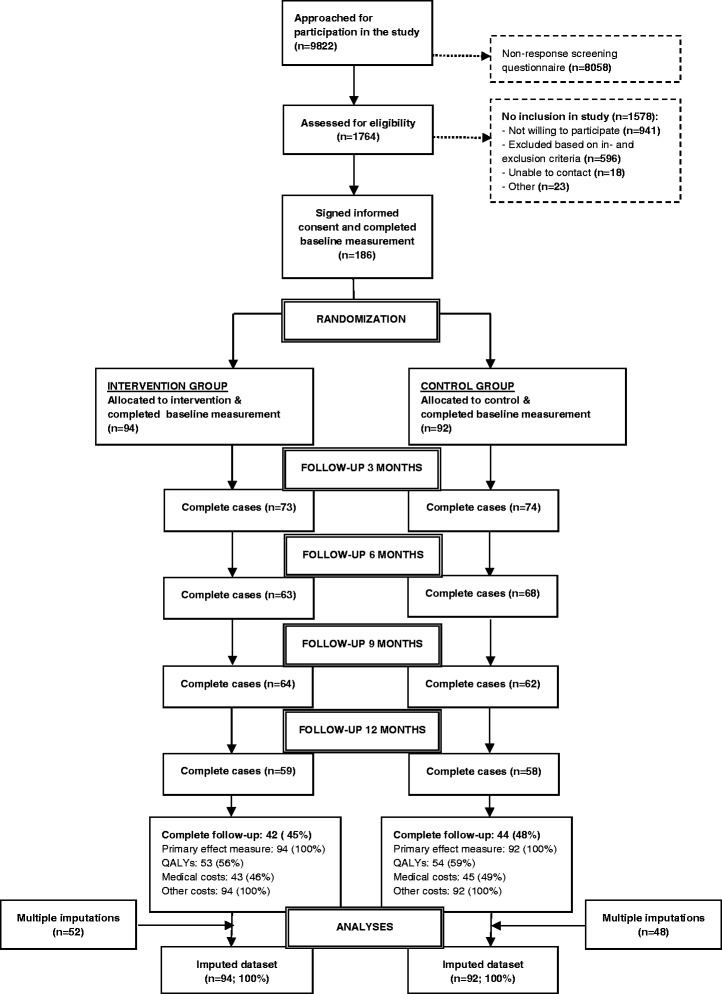
Flow of participants in the participatory supportive RTW program

A total of 186 participants were randomly allocated to the intervention (*n* = 94) or control group (*n* = 92). Fig. 1 illustrates the flow of participants in the Co-WORK study. Data on QALYs were complete for 58% of participants (*n* = 107; 53 intervention group participants and 54 control group participants). Complete data on medical costs was obtained for 47% of participants (*n* = 88; 43 intervention group participants and 45 control group participants). Data on the primary effect measure and all remaining cost categories were complete for all participants. Additional file 1: Table S1 presents the baseline characteristics for intervention and control group participants with complete and incomplete baseline and follow-up data. A relevant difference was found between the RTW expectancy in the intervention and the control group, indicating a more certain expectancy to RTW in the control group. Relevant differences in age and the intention to return to work were found between complete and incomplete cases in both groups. In the control group, more respondents with complete than incomplete data still had an employment contract at baseline.

### Effectiveness

Small and non-statistically significant differences in effects were found between the intervention and control group. In the intervention group, sustainable RTW was reached on average 6.6 days earlier (95%CI = −37.8–24.6) compared with the control group, and the number of QALYs gained was on average 0.01 points lower (95%CI = −0.08–0.06).

### Costs

Additional file 2: Table S2 presents the cost differences between the intervention and control group from the societal perspective. In the corrected model, average costs for OHC consults, total intervention costs, secondary care costs, total medical costs, and total societal costs (excluding absenteeism costs) were significantly higher in the intervention group.

### Societal perspective

#### Cost-effectiveness

**Fig. 2:**
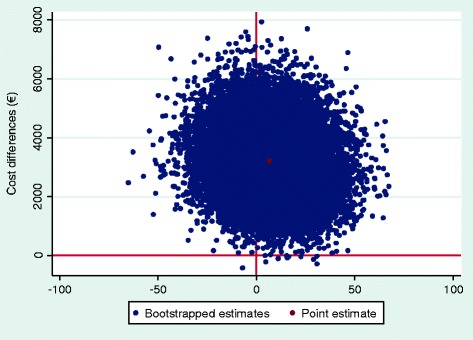
CE-plane for duration until sustainable RTW. CE-plane indicating the uncertainty around the ICER for duration until sustainable RTW (societal perspective)

**Fig. 3:**
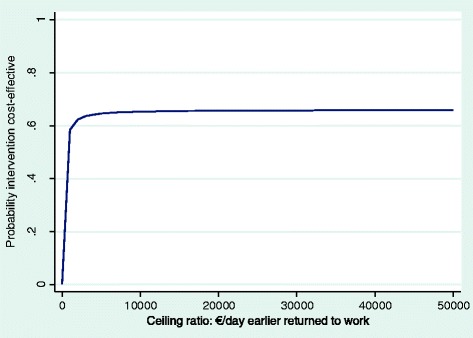
CEAC for duration until sustainable RTW. CEAC indicating the probability of the intervention being cost-effective at different values (€) of willingness to pay per day earlier sustainable RTW (societal perspective)

Additional file 3: Table S3 presents the results of the cost-effectiveness and cost-utility analysis. For duration until sustainable RTW, an ICER of -€487 was found, indicating that a societal investment of €487 was needed per one day earlier sustainable RTW. The majority of incremental CE-pairs (67.3%) was located in the northeast quadrant of the CE-plane (Additional file 3: Table S3, Fig. 2), indicating that the intervention was on average more costly and more effective. The wide distribution of incremental CE-pairs in this plane illustrates a large level of uncertainty around the cost-effectiveness estimate. Figure 3 shows that when the willingness to pay for one day earlier return to work is €0, the probability that the participatory supportive RTW program can be considered cost-effective compared to usual OHC is about zero. This probability increases with an increasing willingness to pay, until it reaches a maximum probability of about 0.64 at a willingness to pay of about €10 000.

#### Cost-utility

**Fig. 4:**
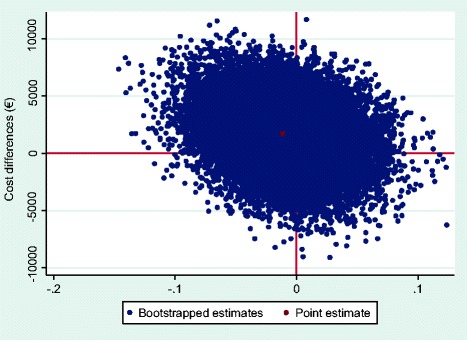
CE-plane for QALYs gained. CE-plane indicating the uncertainty around the ICER for QALYs gained (societal perspective)

**Fig. 5:**
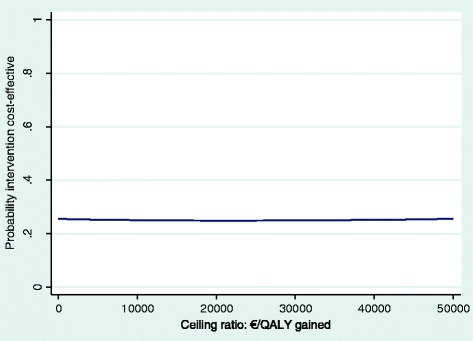
CEAC for QALYs gained. CEAC indicating the probability of the intervention being cost-effective at different values (€) of willingness to pay per QALY gained (societal perspective)

For QALYs, an ICER of -€125 357 was found, indicating that a QALY lost was associated with a societal cost of €125 357. This relatively large negative ICER was the result of a very small difference in QALYs gained between the intervention and control group. The majority of incremental CE-pairs (50.9%) was located in the northwest quadrant of the CE-plane (Additional file 3: Table S3, Fig. 4), indicating that the intervention was on average more costly and less effective. A relatively large level of uncertainty around the cost-utility estimate was visible. Figure 5 illustrates that regardless of the willingness to pay, the maximum probability of the new program being cost-effective compared with usual OHC was about 0.27.

### Social insurer’s perspective

#### Financial return

Additional file 4: Table S4 presents the results of the ROI analysis. The total benefits from the social insurer’s perspective were on average -€784 (95%CI = €-3589–€1819), indicating higher costs for sickness benefit and employment benefit payment in the intervention group compared with the control group. The NB was on average -€1224 (95%CI = −€4048–€1503), suggesting a net loss for the SSA of €1224 per intervention group participant. The BCR was -€1.80 (95%CI = −€9.60–€6.50), which suggests that each Euro invested in the participatory supportive RTW program resulted in a loss of €1.80. The ROI was −278% (95%CI = −1058–548), indicating a loss of 278% per Euro invested. None of these estimates was statistically significant. The estimated maximum probability of financial return was 0.18, indicating a low probability of a positive return on investment.

### Sensitivity analyses

Results of SA2, SA4 and SA5 were similar to those of the main analyses, whereas the outcomes of SA1 and SA3 differed in some aspects from those of the main analyses or contained useful additional information (Additional file 3: Table S3). In SA1 (complete-case analyses), the average difference in total societal costs (excluding absenteeism costs) between both groups was no longer statistically significant. In SA3 (per-protocol analyses), from the societal perspective, a (not statistically significant) longer duration until sustainable RTW was found in the per-protocol group (*n* = 36) compared with the control group, whereas in the main analysis a (not statistically significant) shorter duration was found for the intervention group. Despite these differences, all sensitivity analyses revealed a low probability of cost-effectiveness or financial return, which is in accordance with the main analyses.

## Discussion

This study evaluated the cost-effectiveness, cost-utility, and return on investment of a participatory supportive RTW program aimed at shortening the duration until sustainable RTW of workers without an employment contract, sick-listed due to a CMD, compared with usual OHC. From a societal perspective, the program had no significant effect on the duration until sustainable RTW and QALYs gained. Intervention costs and medical costs were significantly higher in the intervention group, resulting in significantly higher total societal costs. The probability of cost-effectiveness for both outcomes was relatively low (i.e. a maximum probability of cost-effectiveness of 0.64 for the duration until sustainable RTW at a willingness to pay of about €10 000 per day, and a maximum probability of cost-effectiveness of 0.27 for QALYs gained regardless of the willingness to pay). Furthermore, from the social insurer’s perspective there was a low probability of financial return. As such, the present study does not provide evidence to implement this program in the Dutch social security sector for economic reasons.

### Comparison with other studies

Over the past two decades, several economic evaluations of similar participatory RTW programs were conducted among different populations and settings [32–35]. An economic evaluation conducted in a similar setting was the study of Vermeulen et al. [35]. They evaluated a participatory RTW program for temporary agency workers and unemployed workers sick-listed due to musculoskeletal disorders. Their study revealed that a societal investment of €82 was needed per one day earlier sustainable RTW, which was much lower than the ICER found in the present study (i.e. €487/day). Furthermore, high probabilities of cost-effectiveness at a low willingness to pay were found in economic evaluations of a participatory RTW program for employees with low back pain [32, 33]. More in line with our findings were the results from an economic evaluation of a participatory RTW program for employees with distress by van Oostrom et al. [34]. They found a low probability of the program being cost-effective in reducing time to sustainable RTW compared with usual care from the societal perspective. However, for a subgroup of their population with the baseline intention to return to work despite symptoms, the intervention was on average more effective and less costly than usual care. Both the studies of Vermeulen et al. [35] and van Oostrom et al. [34] revealed a low probability of the intervention being cost-effective in terms of QALYs, as was also seen in the present study.

Differences between findings from the present study and the aforementioned studies could be related to differences in effectiveness of the interventions, which have been discussed in more detail in our effectiveness evaluation [7]. Also the large role of implementation failures in the effectiveness of the participatory supportive RTW program, such as the very low number of intervention group participants that had actually started in the participatory supportive RTW program (*n* = 36) and the low protocol adherence within this ‘per-protocol group’, has been discussed in our effectiveness evaluation [7]. The present study, however, provides important new insights into related costs. In the present study the mean costs for applied OHC were highest in the intervention group, but still lower than costs for applied OHC for the intervention group in the study by Vermeulen et al. [35]. These higher costs in the study of Vermeulen et al. seem to be (partly) related to the additional costs needed to realize an early RTW, i.e. costs for rewarding a commercially operating vocational rehabilitation agency, which had been more successful in their study (19 versus nine job placements) [36, 37]. Furthermore, in the present study mean secondary care costs were significantly higher in the intervention group compared to the control group, while in the study of Vermeulen et al. [35] mean secondary care costs were highest in the control group. However, the higher secondary care costs for the intervention group were in line with the findings of Van Oostrom et al. [34]. A post-hoc analysis following our study indicated that in the intervention group during follow-up more participants reported that they had received specialized mental healthcare (*n* = 47; 50%) compared with the control group (*n* = 37; 40.2%), i.e. consultations at an institute for specialized mental healthcare, treatment by a psychologist/ psychiatrists/ psychotherapist, or (part-time) day care for mental health complaints, although differences were not statistically significant and data was not complete for all participants.

### Study implications

Although the findings from our post-hoc analysis should be interpreted with caution, these findings may partly explain the association between allocation to the new participatory supportive RTW program and higher secondary care costs. An early focus on RTW may have placed high demands on both participants and professionals involved, as the most common approach for sick-listed workers with mental health problems is still to ‘train-and-place’ in (sheltered or volunteer) work [1]. Possibly, the prospect of RTW in a competitive job and no longer being entitled to OHC and sickness benefit payment may have increased feelings of insecurity and stress in these participants. Results of our previous qualitative evaluation on the execution of the program in practice also showed that many stakeholders expressed their doubts on the feasibility of this early focus on RTW, and suggested that often an increase of empowerment or mental resilience was first needed [38]. This means that the need for specialized (mental) healthcare possibly became more prominent. Therefore, it may be worthwhile to consider a more intensive and ongoing support by a multidisciplinary team of professionals from the SSA, the (mental) healthcare sector, and from a vocational rehabilitation agency, and a more simultaneously focus on treatment and vocational needs. From an economic perspective, further research is necessary to investigate whether such an approach could reduce secondary care costs without increasing intervention costs.

In addition, for any future intervention for sick-listed workers without an employment contract, it is important to consider how costs needed to realize an early RTW in the absence of a job to return to can remain low. In this regard, Vermeulen et al. [35] proposed several measures, such as realizing subsidized workplaces, increasing responsibilities of employers with regard to facilitation of RTW, and creating a network of potential workplaces. Very recently in the Netherlands, application of a no risk policy for (ex) cancer patients without an employment contract was considered. This policy compensates employers for sickness absence costs, in order to create an incentive for employers to hire particularly these workers [39]. Future research is needed to assess whether such measures may also contribute to a cost-effective RTW program for workers without an employment contract, sick-listed due to a CMD.

### Strengths and limitations

This study provides insight into the costs of the participatory supportive RTW program in relation to its effects. Although the program showed no beneficial or adverse effect on the duration until sustainable RTW, information about its associated costs is needed to determine its probability of cost-effectiveness and financial return. Moreover, reporting the probability of cost-effectiveness and financial return of this program contributes to unbiased systematic reviews on the resource implications of these kind of interventions.

Another strength concerns the use of state-of-the-art statistical measures, i.e. the use of multiple imputations, SUR analyses and bootstrapping. This was one of the first studies in which bootstrapping techniques were not only used to estimate 95% CIs surrounding skewed cost data, but also to estimate the level of uncertainty around NB, BCR and ROI estimates, as well as the probability of financial return, which is very useful for decision-makers in OHC.

A third strength is the study’s pragmatic RCT design, which made it possible to conduct an economic evaluation in the ‘real-life’ setting. A disadvantage of this design is that caution is needed when generalizing the results of this study to another jurisdiction.

A fourth strength concerns the use of registered data by the SSA. Herewith, possible bias caused by self-report, such as recall bias, was minimized. Furthermore, data on RTW, applied OHC and paid benefits by the SSA could be obtained for all participants. Consequently, only five imputed datasets were needed to conduct the analyses. Nevertheless, due to missing self-reported data on QALYs and medical costs, data was complete for only 46% of participants. We dealt with this limitation by using multiple imputations.

Another limitation of this study may be the assessment of medication use. The self-reported data on medication use were often difficult to interpret, as medication names were misspelled or the brand name was used. To limit bias, we chose to only consider the use of psychotropics as these were easy to recognize, and the use of these medications was most likely to be affected by the intervention under study.

A third limitation is that presenteeism during work resumption was not taken into account, although presenteeism costs can be high. However, a sophisticated method for estimating and valuing presenteeism does currently not exist and therefore only a crude estimate of presenteeism costs could have been provided.

For our estimation of productivity loss we did not take into account hours worked in unpaid employment, which was a fourth limitation. This may have led to an overestimation of the actual productivity loss in both groups. However, only self-reported information about work resumption in unpaid labor was available and this was often incomplete. Therefore, the number of hours worked in unpaid employment could not be assessed properly. Moreover, because sick-listed workers without an employment contract are more often low-skilled and have less work experience compared to sick-listed employees [40], they may also be less productive compared to other employees. Therefore, the estimated price of productivity loss for a Dutch worker per hour, based on sex and age that was used to value productivity loss, possibly also resulted in an overestimation of productivity loss. Nevertheless, because an overestimation of productivity loss probably took place in both groups, we were still able to compare productivity loss between these groups.

Finally, a very large number of sick-listed workers was sent an invitation for participation in this study (*N* = 9822) to reach those sick-listed workers with mental health problems, because it was not possible to select potential participants based on a registered mental health problem. This recruitment procedure could be considered as a limitation of this study. We do not know how many sick-listed workers of those who did not respond to the questionnaire (*N* = 8058) actually were sick-listed due to a CMD. Still, we can assume that among this group there were sick-listed workers who actually would have met the criteria for eligibility. The large number of non-responders and the large number of sick-listed workers who responded to the invitation but were not willing to participate (*N* = 941), indicate that selection bias may have played a role. Possibly, sick-listed workers who participated in this study were more willing to (actively) participate in the new program, compared to the ones who did not. The possibility of selection bias could further complicate generalizing the results of this study to other settings, and is therefore an important limitation.

## Conclusions

The participatory supportive RTW program was neither cost-effective in improving sustainable RTW nor in gaining QALYs from the societal perspective. Also, from the perspective of the SSA, the program did not result in a positive financial return. Based on the results of this study, we cannot recommend implementing the participatory supportive RTW program in the Dutch social security sector.

## Additional files

Additional file 1: Table S1. Baseline characteristics. (DOCX 18 kb)
Additional file 2: Table S2. Mean costs per participant in the intervention and control group and crude and adjusted cost differences between both groups during follow-up. (DOCX 16 kb)
Additional file 3: Table S3. Differences in pooled mean costs and effects (95%CIs), ICERs, and the distribution of incremental CE-pairs around the quadrants of the CE-planes (societal perspective). (DOCX 18 kb)
Additional file 4: Table S4. Intervention costs, benefits, NBs, BCRs, and ROIs per participant (social insurer’s perspective). (DOCX 16 kb)

## Abbreviations

ASE: Attitude, Social influence and self-Efficacy
BCA: Bias-corrected and accelerated
BCR: Benefit Cost Ratio
CEA: Cost-effectiveness analysis
CEAC: Cost-effectiveness acceptability curve
CE-pairs: Cost-effect pairs
CE-plane: Cost-effectiveness plane
CIs: Confidence intervals
CMDs: Common mental disorders
CUA: Cost-utility analysis
FCA: Friction Cost Approach
HCA: Human Capital Approach
ICERs: Incremental cost-effectiveness ratios
NB: Net Benefits
NTR: Netherlands Trial Register
OHC: Occupational healthcare
QALYs: Quality-adjusted life years
RCT: Randomized controlled trial
ROI: Return on investment
RTW: Return to work
SA: Sensitivity Analyses
SSA: Social Security Agency
SUR: Seemingly unrelated regression
Tic-P: The Trimbos/iMTA questionnaire for costs associated with psychiatric illness
