# Supplementary material for: A participatory supportive return to work program for workers without an employment contract, sick-listed due to a common mental disorder: an economic evaluation alongside a randomized controlled trial
Source: BMC Public Health. 2017 Feb 2;17:162. doi: 10.1186/s12889-017-4079-0 (PMC5290622; doi:10.1186/s12889-017-4079-0)
Supplement: Additional file 1: Table S1. — Baseline characteristics. (DOCX 18 kb) [file 12889_2017_4079_MOESM1_ESM.docx]

**Additional file 1: Table S1 Baseline characteristics**

*Additional material to article by L. Lammerts, J.M. Van Dongen, F.G. Schaafsma^1^, W. van Mechelen and J.R. Anema ‘A participatory supportive return to work program for workers without an employment contract, sick-listed due to a common mental disorder: an economic evaluation alongside a randomized controlled trial’ in BMC Public Health*

^1^Department of Public and Occupational Health, EMGO+ Institute for Health and Care Research, VU University Medical Center. E-mail: [f.schaafsma@vumc.nl](mailto:f.schaafsma@vumc.nl)

|  | Intervention group | | | Control group | | |
| --- | --- | --- | --- | --- | --- | --- |
| Baseline characteristics | **All**  **(n=94)** | **Complete**  **(n=42)** | **Incomplete**  **(n=52)** | **All**  **(n=92)** | **Complete**  **(n=44)** | **Incomplete**  **(n=48)** |
| *Demographic characteristics:* |  |  |  |  |  |  |
| *Gender, n (%) Female* | 45 (48%) | 21 (49%) | 24 (47%) | 47 (51%) | 23 (51%) | 24 (51%) |
| *Age in years, mean (SD)* | 45.7 (10.6) | 46.8 (9.9) | 44.8 (11.2) | 46.3 (10.0) | 49.0 (9.3) | 43.6 (10.0) |
| *Education*, *n (%) Low ^a^* | 26 (28%) | 11 (26%) | 15 (29%) | 23 (25%) | 13 (29%) | 10 (21%) |
| *Work status:* |  |  |  |  |  |  |
| *Employment contract at baseline, n (%) Yes* | 11 (12%) | 6 (14%) | 5 (10%) | 14 (15%) | 2 (4%) | 12 (26%) |
| *Type of worker before reporting sick:*  N (%) unemployed worker  N (%) temporary agency worker  N (%) fixed-term contract worker | 88 (94%)  4 (4%)  2 (2%) | 40 (93%)  1 (2%)  2 (5%) | 48 (94%)  3 (6%)  0 (0%) | 85 (92%)  2 (2%)  5 (5%) | 44 (98%)  0 (0%)  1 (2%) | 41 (87%)  2 (4%)  4 (9%) |
| *Work schedule in last job, n (%) day work* | 72 (77%) | 31 (72%) | 41 (80%) | 75 (82%) | 38 (84%) | 37 (79%) |
| *Working hours per week in last job, mean (SD)* | 32.6 (11.6) | 32.0 (10.8) | 33.0 (12.3) | 31.4 (10.8) | 31.5 (9.8) | 31.2 (11.8) |
| *Years worked in last job, mean (SD)* | 10.0 (10.0) | 11.0 (10.9) | 9.1 (9.2) | 8.7 (9.6) | 8.7 (9.8) | 8.6 (9.5) |
| *Expectation regarding ability for full RTW in 6 months, n (%) (very) certain* | 4 (4%) | 1 (2%) | 3 (6%) | 16 (17%) | 8 (18%) | 8 (17%) |
| *ASE, mean (SD): ^b^*  *Intention to RTW, n (%) Yes*  *Attitude (6-30 score)*  *Normative beliefs (4-20 score)*  *Social modelling (2-10 score)*  *Self-efficacy (2-10 score)* | 78 (83%)  15.7 (5.2)  12.0 (3.1)  4.8 (1.9)  6.3 (1.8) | 39 (91%)  15.2 (4.4)  11.7 (2.9)  4.6 (1.4)  6.0 (1.4) | 39 (77%)  16.0 (5.9)  12.3 (3.3)  5.0 (2.2)  6.6 (2.1) | 81 (88%)  14.9 (4.2)  12.6 (2.6)  4.8 (1.5)  6.2 (1.6) | 42 (93%)  14.1 (3.8)  12.6 (2.1)  4.9 (1.4)  6.2 (1.6) | 39 (83%)  15.8 (4.4)  12.6 (3.0)  4.7 (1.7)  6.3 (1.6) |
| *Fear avoidance beliefs (4-40 score), mean (SD)* ^c^ | 29.0 (6.9) | 29.1 (6.7) | 28.8 (7.0) | 28.5 (7.0) | 28.9 (6.4) | 28.2 (7.7) |

*^a^* Low educational level included no education, primary school or lower vocational education

^b^ A lower score on these scales corresponds with a more positive attitude regarding RTW (attitude), a stronger belief that other people think work resumption is important (normative beliefs), finding it more important what other people think (social modelling) and a stronger feeling of self-efficacy regarding RTW (self-efficacy)

^c^ A higher score on this scale, indicates a stronger belief that health complaints could interfere with RTW.
