# Supplementary material for: A participatory supportive return to work program for workers without an employment contract, sick-listed due to a common mental disorder: an economic evaluation alongside a randomized controlled trial
Source: BMC Public Health. 2017 Feb 2;17:162. doi: 10.1186/s12889-017-4079-0 (PMC5290622; doi:10.1186/s12889-017-4079-0)
Supplement: Additional file 2: Table S2. — Mean costs per participant in the intervention and control group and crude and adjusted cost differences between both groups during follow-up. (DOCX 16 kb) [file 12889_2017_4079_MOESM2_ESM.docx]

**Additional file 2: Table S2 Mean costs per participant in the intervention and control group and crude and adjusted cost differences between both groups during follow-up**

*Additional material to article by L. Lammerts, J.M. Van Dongen, F.G. Schaafsma^1^, W. van Mechelen and J.R. Anema ‘A participatory supportive return to work program for workers without an employment contract, sick-listed due to a common mental disorder: an economic evaluation alongside a randomized controlled trial’ in BMC Public Health*

^1^Department of Public and Occupational Health, EMGO+ Institute for Health and Care Research, VU University Medical Center. E-mail: [f.schaafsma@vumc.nl](mailto:f.schaafsma@vumc.nl)

| **Cost category** | **Intervention**  **n=94; mean (SEM)** | **Control**  **n=92; mean (SEM)** | **Mean cost difference**  **Model 1^a^**  **(95%CI)** | **Mean cost difference**  **Model 2^b^**  **(95%CI)** |
| --- | --- | --- | --- | --- |
| Intervention costs | 1130 (108) | 623 (84) | 507 (252 to 789) | 440 (143 to 734) |
| OHC consults | 420 (36) | 305 (22) | 114 (39 to 205) | 135 (57 to 255) |
| Additional support | 557 (101) | 318 (77) | 239 (-1 to 507) | 151 (-111 to 418) |
| Training costs | 153 (NA) | 0 (NA) | 153 (NA) | 153 (NA) |
| Medical costs | 6323 (946) | 3452 (486) | 2871 (1073 to 5147) | 2765 (918 to 4876) |
| Primary care | 1858 (199) | 1649 (185) | 210 (-251 to 648) | 179 (-277 to 675) |
| Secondary care | 4432 (875) | 1769 (432) | 2662 (1007 to 4740) | 2587 (879 to 4510) |
| Medication | 34 (5) | 34 (5) | -1 (-14 to 12) | -2 (-15 to 11) |
| Absenteeism costs | 44413 (1801) | 44981 (1736) | -569 (-5185 to 4472) | -549 (-5185 to 4472) |
| **Total societal costs** | **51866 (2076)** | **49057 (1884)** | **2809 (-2451 to 8385)** | **1712 (-3520 to 6650)** |
| **Total societal costs (excluding absenteeism costs)** | **7453 (956)** | **4075 (496)** | **3378 (1557 to 5670)** | **3206 (1346 to 5337)** |

Abbreviations: n=number, CI=Confidence Interval, SEM=Standard Error of the Mean

^a^ Not corrected for confounders

^b^ Corrected for baseline differences in demographic characteristics + type of worker + RTW expectation + ASE + intention to RTW + fear avoidance beliefs
