# Supplementary material for: A participatory supportive return to work program for workers without an employment contract, sick-listed due to a common mental disorder: an economic evaluation alongside a randomized controlled trial
Source: BMC Public Health. 2017 Feb 2;17:162. doi: 10.1186/s12889-017-4079-0 (PMC5290622; doi:10.1186/s12889-017-4079-0)
Supplement: Additional file 3: Table S3. — Differences in pooled mean costs and effects (95%CIs), ICERs, and the distribution of incremental CE-pairs around the quadrants of the CE-planes (societal perspective). (DOCX 18 kb) [file 12889_2017_4079_MOESM3_ESM.docx]

**Additional file 3.**

*Additional material to article by L. Lammerts, J.M. Van Dongen, F.G. Schaafsma^1^, W. van Mechelen and J.R. Anema ‘A participatory supportive return to work program for workers without an employment contract, sick-listed due to a common mental disorder: an economic evaluation alongside a randomized controlled trial’ in BMC Public Health*

^1^Department of Public and Occupational Health, EMGO+ Institute for Health and Care Research, VU University Medical Center. E-mail: [f.schaafsma@vumc.nl](mailto:f.schaafsma@vumc.nl)

**Table A3. Differences in pooled mean costs and effects (95%CIs), ICERs, and the distribution of incremental CE-pairs around the quadrants of the CE-planes (societal perspective)**

| **Analysis** | **Sample size** | | **Effect measures** | **∆C (95% CI)** | **∆E (95%CI)** | **ICER** | **Distribution CE-plane (%)** | | | |
| --- | --- | --- | --- | --- | --- | --- | --- | --- | --- | --- |
|  | **Intervention** | **Control** |  | **€** | **Points** | **€/point** | **NE^1^** | **SE^2^** | **SW^3^** | **NW^4^** |
| **Main analysis** – *Imputed dataset* | 94 | 92 | Duration to sustainable RTW (days) | 3206 (1346 to 5337) | -6.6 (-37.8 to 24.6) | -487 | 67.3 | 0.0 | 0.0 | 32.7 |
|  | 94 | 92 | QALY | 1712 (-3520 to 6650) | -0.01 (-0.08 to 0.06) | -125357 | 28.9 | 12.4 | 12.8 | 50.9 |
| **SA1** – *Complete-case analysis* | 42 | 44 | Duration to sustainable RTW (days) | 2261 (-500 to 6028) | -17 (-56 to 21) | -129 | 77.3 | 6.5 | 1.2 | 15.0 |
|  | 42 | 44 | QALY | 97 (-7195 to 6817) | 0.07 (-0.02 to 0.16) | 1377 | 45.9 | 47.2 | 2.3 | 4.5 |
| **SA2** – *Excluding healthcare outliers* | 89 | 90 | Duration to sustainable RTW (days) | 2657 (1195 to 4537) | -3.6 (-36 to 28) | -738 | 59.9 | 0.0 | 0.0 | 40.1 |
|  | 89 | 90 | QALY | 1762 (-3364 to 6821) | -0.02 (-0.09 to 0.05) | -76267 | 19.3 | 9.8 | 15.1 | 55.8 |
| **SA3 –** *Per-protocol* | 36 | 92 | Duration to sustainable RTW (days) | 3355 (723 to 6403) | 3.1 (-40.5 to 46.7) | 1082 | 41.5 | 0.9 | 0.6 | 57.0 |
|  | 36 | 92 | QALY | 3798 (-4126 to 10973) | 0.01 (-0.07 to 0.09) | 410061 | 45.5 | 12.4 | 3.8 | 38.4 |
| **SA4** – *FCA; 23 weeks* | 94 | 92 | QALY | 2606 (98 to 5317) | -0.01 (-0.08 to 0.06) | -215336 | 35.0 | 1.3 | 1.1 | 62.7 |
| **SA5** – *FCA; 12 weeks* | 94 | 92 | QALY | 3160 (1145 to 5381) | -0.01 (-0.08 to 0.06) | -269932 | 36.1 | 0.0 | 0.0 | 63.7 |

Abbreviations: C=Costs, E=Effects, ICER= Incremental Cost-Effectiveness Ratio, CE-plane=Cost-Effectiveness-plane, QALYs=Quality Adjusted Life Years

Note: cost and effect differences were corrected for baseline differences in demographic characteristics + type of worker + RTW expectation + ASE + intention to RTW + fear avoidance beliefs.

^1^ Refers to the northeast quadrant of the CE-plane, indicating that the intervention is more effective and more costly usual care

^2^ Refers to the southeast quadrant of the CE-plane, indicating that the intervention is more effective and less costly than usual care

^3^ Refers to the southwest quadrant of the CE-plane, indicating that the intervention is less effective and less costly than usual care

^4^ Refers to the northwest quadrant of the CE-plane, indicating that the intervention is less effective and more costly than usual care
