# Supplementary material for: A participatory supportive return to work program for workers without an employment contract, sick-listed due to a common mental disorder: an economic evaluation alongside a randomized controlled trial
Source: BMC Public Health. 2017 Feb 2;17:162. doi: 10.1186/s12889-017-4079-0 (PMC5290622; doi:10.1186/s12889-017-4079-0)
Supplement: Additional file 4: Table S4. — Intervention costs, benefits, NBs, BCRs, and ROIs per participant (social insurer’s perspective). (DOCX 16 kb) [file 12889_2017_4079_MOESM4_ESM.docx]

**Additional file 4: Table S4. Intervention costs, benefits, NBs, BCRs, and ROIs per participant (social insurer’s perspective)**

*Additional material to article by L. Lammerts, J.M. Van Dongen, F.G. Schaafsma^1^, W. van Mechelen and J.R. Anema ‘A participatory supportive return to work program for workers without an employment contract, sick-listed due to a common mental disorder: an economic evaluation alongside a randomized controlled trial’ in BMC Public Health*

^1^Department of Public and Occupational Health, EMGO+ Institute for Health and Care Research, VU University Medical Center. E-mail: [f.schaafsma@vumc.nl](mailto:f.schaafsma@vumc.nl)

| **Analysis** | **Sample size** | | **Costs** | **Benefits** | **Financial return** | | | |
| --- | --- | --- | --- | --- | --- | --- | --- | --- |
|  | **Intervention** | **Control** | **Total (95% CI)** | **Total (95% CI)** | **NB^1^ (95% CI)** | **BCR^2^ (95% CI)** | **ROI (%)^3^ (95% CI)** | **Probability** |
| **Main analysis** -  *Imputed dataset* | 94 | 92 | 440 (143 to 734) | -784 (-3589 to 1819) | -1224 (-4084 to 1503) | -1.8 (-9.6 to 6.5) | -278 (-1058 to 548) | 0.18 |
| **SA1** – *Complete-case analysis* | 92 | 90 | 423 (128 to 737) | -920 (-3755 to 1846) | -1343 (-4262 to 1527) | -2.2 (-10.9 to 6.7) | -317 (-1191 to 569) | 0.17 |
| **SA2** – *Excluding healthcare outliers* | 89 | 90 | 432 (119 to 730) | -1032 (-3766 to 1865) | -1464 (-4357 to 1393) | -2.4 (-10.9 to 5.7) | -339 (-1190 to 469) | 0.16 |
| **SA3** *– Per-protocol* | 36 | 92 | 1121 (700 to 1554) | -1654 (-5003 to 1560) | -2775 (-6229 to 606) | -1.4 (-4.5 to 1.6) | -248 (-553 to 63) | 0.05 |

Abbreviations: CI: Confidence Interval, NB: Net Benefit, BCR: Benefit Cost Ratio, ROI: Return-On-Investment, I: Intervention, C: Control,

Note1: Costs and benefits were corrected for baseline differences in demographic characteristics + type of worker + RTW expectation + ASE + intention to RTW + fear avoidance beliefs

Note2: Financial returns are positive if the following criteria are met: NB>0, BCR>1, and ROI>0

^1^ Indicates the amount of money returned after intervention costs are recovered

^2^ Indicates the amount of money returned per Euro invested in the intervention

^3^ Indicates the percentage of profit per Euro invested in the intervention
